# Hydrogen-induced nanotunnel opening within semiconductor subsurface

**DOI:** 10.1038/ncomms3800

**Published:** 2013-11-21

**Authors:** Patrick Soukiassian, Erich Wimmer, Edvige Celasco, Claudia Giallombardo, Simon Bonanni, Luca Vattuone, Letizia Savio, Antonio Tejeda, Mathieu Silly, Marie D’angelo, Fausto Sirotti, Mario Rocca

**Affiliations:** 1 Istituto Materiali per Elettronica e Magnetismo—CNR, 16146 Genova, Italy; 2 Commissariat à l’Energie Atomique et aux Energies Alternatives, SIMA, DSM-IRAMIS-SPCSI, Saclay, Bât. 462, 91191 Gif sur Yvette, France; 3 Synchrotron SOLEIL, L’Orme des Merisiers, Saint-Aubin, 91192 Gif sur Yvette, France; 4 Materials Design, Inc., Santa Fe, New Mexico 87501, USA; 5 Materials Design, SARL, 92120 Montrouge, France; 6 Dipartimento di Fisica dell’Università di Genova, 16146 Genova, Italy; 7 Institut Jean Lamour, CNRS-Université de Lorraine, 54506 Vandoeuvre les Nancy, France; 8 Institut des NanoSciences de Paris, CNRS UMR 7588, Université Pierre et Marie Curie, 75251 Paris, France; 9 *Present address*: SwissLitho AG, Technoparkstrasse 1, 8005 Zurich, Switzerland

## Abstract

One of the key steps in nanotechnology is our ability to engineer and fabricate low-dimensional nano-objects, such as quantum dots, nanowires, two-dimensional atomic layers or three-dimensional nano-porous systems. Here we report evidence of nanotunnel opening within the subsurface region of a wide band-gap semiconductor, silicon carbide. Such an effect is induced by selective hydrogen/deuterium interaction at the surface, which possesses intrinsic compressive stress. This finding is established with a combination of *ab-initio* computations, vibrational spectroscopy and synchrotron-radiation-based photoemission. Hydrogen/deuterium-induced puckering of the subsurface Si atoms marks the critical step in this nanotunnel opening. Depending on hydrogen/deuterium coverages, the nanotunnels are either metallic or semiconducting. Dangling bonds generated inside the nanotunnel offer a promising template to capture atoms or molecules. These features open nano-tailoring capabilities towards advanced applications in electronics, chemistry, storage, sensors or biotechnology. Understanding and controlling such a mechanism open routes towards surface/interface functionalization.

## Abstract (web summary)

Silicon carbide surfaces offer many interesting properties induced by surface strain relief. Soukiassian *et al.* report hydrogen-induced self-organized nano-voids below a silicon carbide surface, and suggest the resultant nanotunnel may be used as a template to capture atoms or molecules.

## Graphical abstract

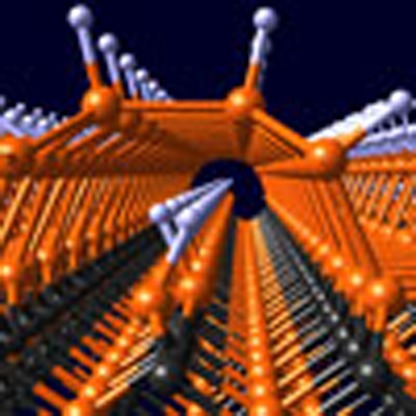

Silicon carbide (SiC) offers fascinating structural, thermomechanical, electronic and chemical properties with a vast range of advanced applications including high-power, high-frequency and high-temperature electronic devices and sensors^1^,^2^,^3^. In addition, SiC has a remarkable biocompatibility making it useful for biomedical applications^4^. Furthermore, it is a substrate especially suitable for the growth of epitaxial graphene with very promising potential applications in electronics and spintronics^5^. SiC exists in >170 polytypes including cubic (3C or *β*), hexagonal and rhombohedral forms^1^. Together with hydrogen and oxygen, Si and C, including SiC, are among the most abundant species in the universe, primarily (80%) composed of the 3C–SiC polytype. Indeed, at the end of the nineteenth century, Henri Moissan found SiC on a meteorite in the Diablo Canyon, Arizona^6^. The cubic 3C-SiC(001) surface is of special interest with its atomic structure and many of its properties driven by surface strain/stress relief^7^,^8^,^9^,^10^,^11^,^12^,^13^,^14^,^15^,^16^,^17^,^18^,^19^,^20^,^21^. This leads to a large number of surprising and interesting surface arrangements with more than ten different well-characterized surface reconstructions (such as 3 × 2, 8 × 2, 5 × 2, 7 × 2, …, (2*n*+1) × 2; c(4 × 2), c(2 × 2)), ranging from Si-rich to C-rich systems including notably in the latter case, graphene^7^,^8^,^9^,^10^,^11^,^12^,^13^,^14^,^15^,^16^,^17^,^18^,^19^,^20^,^21^,^22^. The atomic structures of these reconstructions are now well understood and established on the ground of advanced experimental techniques such as scanning tunneling microscopy (STM), synchrotron-radiation-based photoemission spectroscopy/diffraction (SR-PES, SR-PED), grazing incidence X-ray diffraction, low-energy electron diffraction, near-edge absorption fine structure and state-of-the-art *ab-initio* calculations^7^,^8^,^9^,^10^,^11^,^12^,^13^,^14^,^15^,^16^,^17^,^18^,^19^,^20^,^21^,^22^. It is therefore important to deepen our understanding of this material so that its full potential can be realized. For these reasons, SiC and its surfaces have attracted special attention and a range of exciting and unique properties have been discovered. Among many others, the self-formation of highly stable massively parallel passive or active atomic lines and nanowires at the surface^7^,^15^,^16^,^17^, and the interaction of H/D atoms with the Si-rich 3C-SiC(001)-3 × 2 surface reconstruction are of special interest^23^,^24^,^25^,^26^,^27^,^28^,^29^,^30^,^31^,^32^,^33^, with the first example of H/D-induced metallization of a semiconductor surface^23^,^26^.

The atomic structure of the Si-rich 3C-SiC(001)-3 × 2 surface reconstruction is now very well understood and established on the basis of atom-resolved real-space STM^8^, synchrotron radiation-based grazing incidence X-ray diffraction^9^ and PED^10^ experiments, and *ab-initio* calculations^11^. The structure includes three Si atomic layers at 1/3, 2/3 and 1 ML, respectively, on top of the first C atomic plane^7^,^8^,^9^,^10^,^11^ as illustrated in Fig. 1. The first plane (1/3ML) includes dimer rows having asymmetric Si–Si dimers all tilted along the same direction, the second plane (2/3 ML) also includes dimer rows having alternating lengths and the third plane also has dimer rows^7^,^8^,^9^,^10^. This complex structure tends to reduce the surface strain. Notice that the H(D)-induced metallization of the 3C-SiC(001)-3 × 2 surface takes place while its initial 3 × 2 array is maintained^23^,^26^.

The present investigation provides evidence of ‘nanotunnel’ formation. Unlike atomic lines or nanowires that are self-grown on a surface^15^,^16^,^17^, such a nanostructure is not generated by self-assembling of atoms, but by the auto-organization of nano-voids below the surface. This void structure is induced by the adsorption of atomic hydrogen/deuterium (H/D) on a 3C-SiC(001)-3 × 2 reconstructed surface. The critical effect, which opens such nanotunnels, occurs by H-induced puckering of Si atoms in the subsurface layers connected with stress relief, thereby decoupling the second Si layer and activating the third Si layer below the surface. These nano-scale void structures are well-oriented and periodically spaced, offering unique opportunities for surface functionalization. In the literature, there are examples of voids generated at a surface or below. However, they are in the μm or sub-μm or mesoscopic scales and exhibit no ordering or self-organization mechanisms, since they are generated by experimental techniques such as ion bombardment or etching^34^,^35^,^36^,^37^. Nano-cavities generated by ion bombardment of a copper surface are, so far, the only voids known at the nanometre scales, but again, they exhibit no spatial ordering^34^.

In this work, we use high-resolution electron energy loss spectroscopy (HREELS) and synchrotron-radiation-based photoemission spectroscopy (SR-PES) combined with *ab-initio* calculations to reveal the opening of nanotunnels induced by H(D) exposures of a 3C-SiC(001)-3 × 2 surface. Nanotunnel opening activates the Si atoms in the third layer inside the tunnel. H atoms binding to these Si atoms transform the system from a semiconducting to a metallic state. Further exposure returns the system to a semiconducting status. The nanotunnel structure is consistent with results from SR-PES, HREELS and earlier infrared absorption spectroscopy (IRAS), STM and SR-based core-level PES results^23^,^24^,^30^.

## Results

### Atomic structure and electronic properties by *ab-initio* simulations

**Figure 1:**
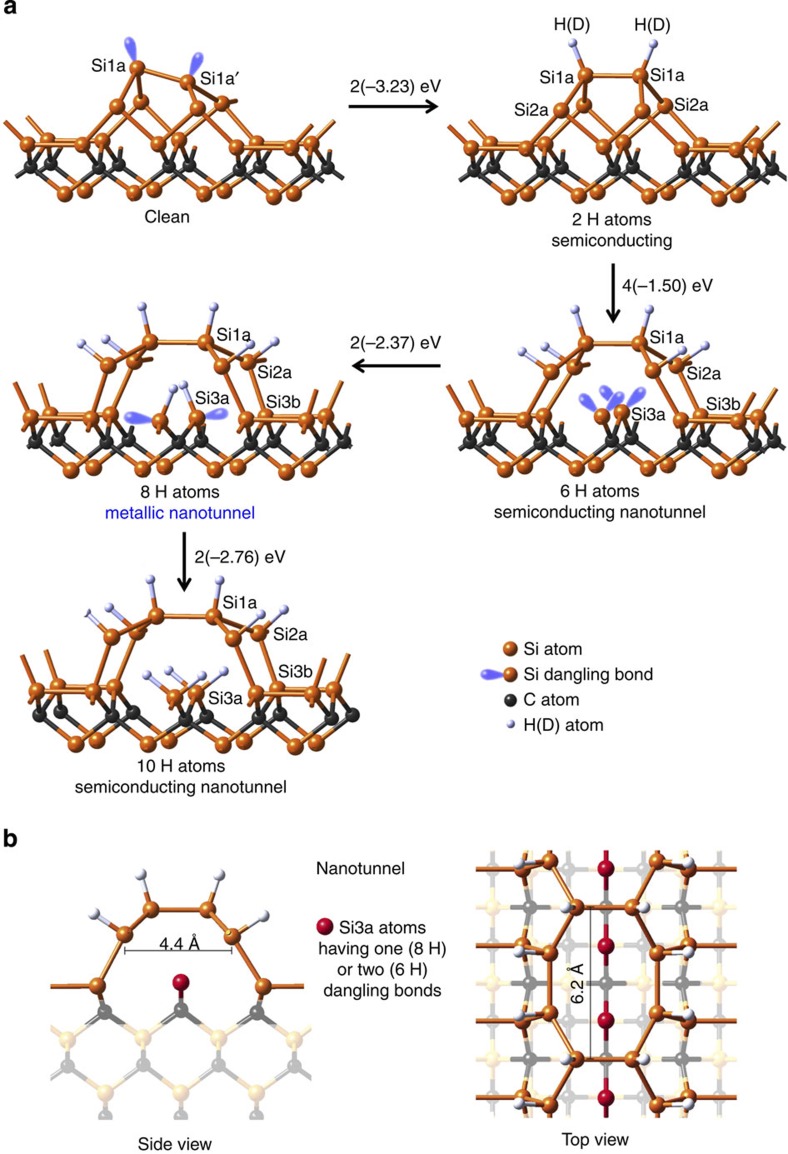
Structures of the clean and H/3C-SiC(100)-3 × 2 surfaces and views of a nanotunnel. (**a**) The 2 H, 6 H, 8 H and 10 H structures refer to the number of H atoms per surface dimer. Nanotunnel opening is seen for 6 H, 8 H and 10 H. The energy release for each step is indicated, as, for example, 2(−3.23) eV, that is, 3.23 eV per H atom. Si1a(b), Si2a and Si3a(b) refer to Si atoms in the first, second and third plane, respectively. Si dangling bonds are depicted in blue. (**b**) The nanotunnel has a size of 6.2 × 4.4 Å (at the level of the second layer). The red atoms within the nanotunnel mark Si atoms with one or two dangling bonds corresponding to the 8 H (metallic) and 6 H (insulating) structures, respectively.

**Figure 2:**
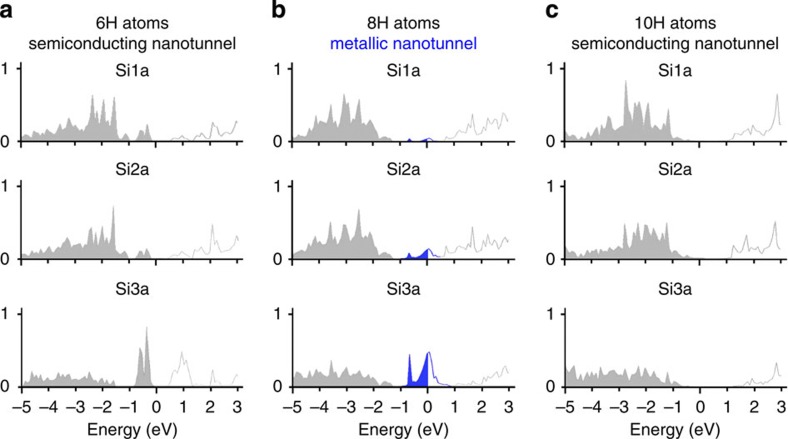
Atom-projected DOS for nanotunnels. The atom-projected DOS are displayed for the nanostructures corresponding to the (**a**) 6 H, (**b**) 8 H and (**c**) 10 H nanotunnels shown in Fig. 1a. For the metallic 8 H nanotunnel (**b**), we notice the DOS build-up at the Fermi Level *E*_F_ (shown in blue), which is occuring predominantly in the third Si layer for the Si3a atoms. Note also the semiconducting-metallic-semiconducting transition upon increasing H coverage, with H atom-induced partially empty Si dangling bonds (shown in blue in Fig. 1) for the metallic structure.

We first look at the results of *ab-initio* calculations for the 3C-SiC(001)-3 × 2 hydrogenation as illustrated in Figs 1 and 2. Starting with the reconstructed 3 × 2 clean surface, H(D) atoms first bind to the Si dangling bonds in the first layer, denoted by Si1a and Si1a′ in Fig. 1 and seen by STM^23^. The bonding is strongly exothermic releasing 3.23 eV per H atom. The resulting structure, labelled as ‘2 H atoms’ is characterized by a symmetric surface dimer with the dangling bonds saturated by H(D) atoms, in excellent agreement with STM results^23^. In the next step, H atoms are placed in the grooves between the dimers and the atom positions are relaxed by energy minimization. The calculations show that the Si atoms in the second layer, labelled Si2a, trap H atoms with an energy release of 1.50 eV per atom. This step breaks the bonds between Si atoms in the second and third layer (Si2a and Si3a), leading to a puckering around the Si2a atoms, and the opening of a nanotunnel. This rearrangement produces reactive Si atoms in the third layer (Si3a). The unsaturated bonds of the Si3a atoms give rise to a double peak in the density of states (DOS) between 0 and −1 eV as shown for the 6 H structure in Fig. 2. The surface remains semiconducting. The formation of the nanotunnel opens the access to the third-layer Si3a atoms while sterically hindering the path to the Si3b atoms. Hence, additional H atoms are likely to bind to the empty dangling bond of the Si3a atoms in the third layer, forming a bonding arrangement H–Si–C–, since the third-layer Si atoms are bonded to C atoms. A partial saturation of the Si3a atoms with H atoms causes a partially filled band at the Fermi level (see blue parts of the DOS in Fig. 2). In other words, at this H-coverage the surface appears to be metallic. Additional exposure to H saturates all dangling bonds of the Si3a atoms and the surface returns to a semiconducting state.

As will be discussed in the following, the computed vibrational frequencies for these configurations as well as the semiconducting–metallic–semiconducting sequence upon increasing H exposure are perfectly consistent with our experimental results for vibrational and electronic properties.

### Vibrational properties measured by HREELS

**Figure 3:**
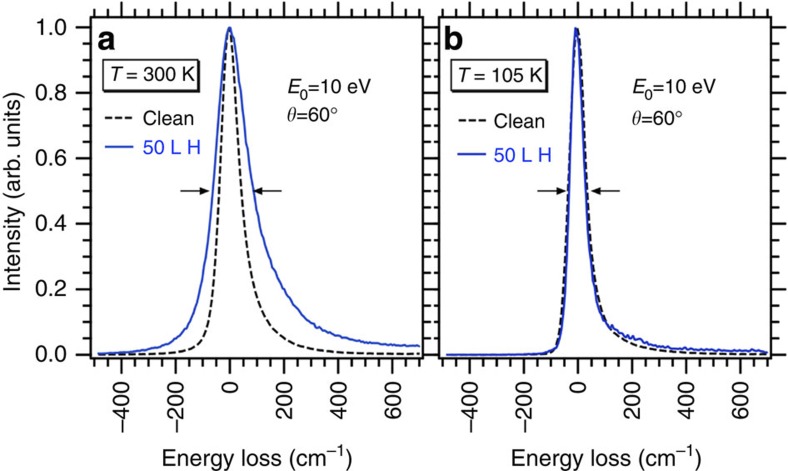
HREELS specular elastic peak for clean and 50L H exposed 3C-SiC(001)-3 × 2. (**a**) HREELS spectra recorded at a temperature of 300 K and (**b**) at a temperature of 105 K. The factor 2 broadening observed at 300 K for the hydrogenated surface is indicative of surface metallization. Such broadening is not present at 105 K in agreement with the suppression of the mechanisms of either multiple excitation of a low frequency plasmon or Drude damping.

Next, we look at the HREELS measurements. Compared with IRAS, HREELS extends over a wider frequency range and can be performed at low sample temperatures, offering the opportunity to explore the low frequency modes predicted by theory. Figure 3a,b display the elastic peaks for the clean and 50L (1L=1 Langmuir=10^−6  ^torr s) H-exposed 3C-SiC(001)-3 × 2 surfaces at 300 and 105 K, respectively. The elastic peak full-width-at-half-maximum of the H-exposed surface is broadened^38^. This broadening is significantly larger at 300 K than at 105 K, indicating either unresolved multiple low frequency plasmon excitation or the onset of a Drude damping^39^ (see also the HREELS experiments section in Methods). In both cases, this means that the 3C-SiC(001)-3 × 2 surface exposed to 50L H has a metallic character.

**Figure 4:**
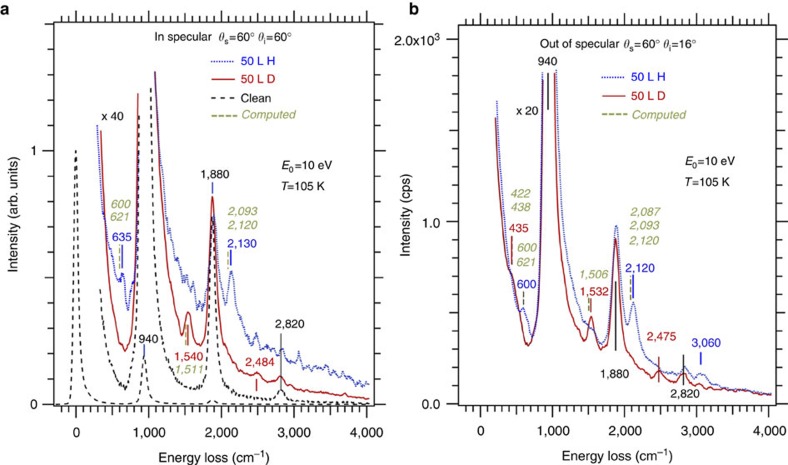
HREELS and computed vibrational frequencies for H/D-induced metallic nanotunnel (**a**) HREELS spectra recorded at a temperature *T*=105 K for **H** (blue dots) and **D** (red continuous line) in specular and (**b**) out of specular conditions. The spectrum for the clean surface (black dash) is shown for comparison, too. Note the combination band between the FK and **H**(**D**) stretch at 3040, cm^−1^ (2,460 cm^−1^). The spectra correspond to 50L **H**(**D**) exposed 3C-SiC(001)-3 × 2 surfaces. The vibrational frequencies measured by HREELS for **H** and **D** are marked by 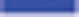 and 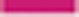 bars, and labelled in blue and red respectively, whereas the corresponding calculated frequencies are labelled in green and marked by a 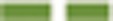 bar. Vibrational frequencies for the clean 3C-SiC(001)-3 × 2 surface are labeled in black and marked by a 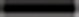 bar.

We now compare the vibrational frequencies measured by HREELS for the 50L H(D)/3C-SiC(100)-3 × 2 surface to those computed for the 8 H metallic model that are displayed in Table 1 together with previous IRAS^23^ measurements. We also investigated the deuterated system to explore regions hidden by the Fuchs–Kliewer (FK) modes. The corresponding vibrational spectra for H(D) are displayed in Fig. 4a,b for in and out of specular conditions, respectively, providing the description of the modes—see Table 1 also listing previous IRAS results^23^. Measurements were performed at 300, 105 and 30 K obtaining very similar results. The losses at 940, 1,880 and 2,820 cm^−1^, correspond to single and multiple excitations of the SiC FK mode^39^, and are also present on the bare surface. The smaller losses at 635 cm^−1^, moving at 600 cm^−1^ out of specular (435 cm^−1^ out of specular only) and at 2,130 cm^−1^, moving to 2,120 cm^−1^ (1,540–1,532 cm^−1^ for the deuterated surface), are due to the bending and stretching of the H(D)–Si bonds, respectively. The latter region had been previously explored also with IRAS at room temperature only, finding compatible results^23^.

These spectral features are found to be in remarkable agreement with theory accounting for all observable modes involving H(D) vibrations. The measured frequency of 635(435) cm^−1^ is associated with a symmetric wagging mode of the Si2a–H(D) group, possibly with an admixture of the wagging mode of the Si1a–H(D) atoms. Owing to the inclination of the Si2a–H(D) bond towards the surface plane, the wagging mode has a strong component perpendicular to the surface and thus is observable by HREELS, while the wagging of the Si1a–H(D) is predominantly parallel to the surface resulting in a much weaker signal. The computed frequencies of 600 (438) cm^−1^ for Si2a–H(D) and 621 (422) cm^−1^ for the Si1a–H(D) modes agree with experiments within the accuracy of vibrational modes obtained by density functional theory (DFT)–generalized gradient approximation and the harmonic approximation. In fact, anharmonicities can change frequencies upto ~80 cm^−1^ for groups such as surface silanols^40^, which provides an indication of the accuracy of the present calculations. As evident from Table 1, the largest difference between experimental and theoretical values is 37 cm^−1^, with the theoretical value always lower than the experimental one, indicating the systematic nature of this error.

Two high frequency modes have been observed with IRAS at 2,140 and 2,118 cm^−1^ (ref. 23). The upper frequency mode has been associated with a Si–H stretch for Si bonded to C, whereas the lower mode is related to H on the topmost Si dangling bond^23^. The former has a higher frequency due to charge transfer from Si to C, which slightly enhances the ionic character of the Si(*δ*^+^)–H(*δ*^−^) bond compared with a Si–Si–H bonding arrangement as it is the case for the Si1a–H bond, in agreement with the higher electron affinity of C compared with Si. The separation of the two modes by 22 cm^−1^ is very well captured by the present calculations for the 8 H structure, which show modes at 2,120 and 2,093 cm^−1^, whereas HREELS gives 2,130 (1,540) cm^−1^ for the symmetric stretch of the surface dimer, which may be influenced by signals from the third layer. Off-specular HREELS data may provide insights on the anti-symmetric stretch modes too, and show indeed slightly lower frequencies. In agreement with experiment, the computations give a lower frequency for the anti-symmetric modes compared with the symmetric stretch ones of the Si1a–H bond. The computed Si2a–H(D) stretch mode at 2,020 (1,457) cm^−1^ is not observed owing to its weak out-of-plane component. Thus, all observed H(D) experimental modes are accounted for by the vibrational properties of 8 H structure. One should note that the modes associated with atoms Si1a and Si2a of the 10 H structure are similar to those of 8H. No modes are present at the frequencies of 778 cm^−1^ for D-exposed or 1,450 cm^−1^ for H-exposed 3C–SiC surface as predicted by some of the DFT calculations for vibrations involving H(D) in bridge positions in the third plane^27^,^28^,^29^. Modes at 1,100 cm^−1^ (H) and 1,025 cm^−1^ (D) are undetectable due to the overlap with the tail of the strong FK mode at 940 cm^−1^. In particular, we note that the corresponding vertically polarized mode at 1,450 cm^−1^ would be easy to observe since it would nicely fall in-between the FK peaks, which is not the case. This excludes H(D) atoms in a bridge bond configuration as a suitable model to explain the metallicity as suggested in some DFT calculations^27^,^28^,^29^. Extended to low crystal temperatures, our HREELS investigation demonstrates no subsequent structural change. Also, the H interaction with the second layer has been invoked in refs 29, 31, 32. A recent calculation suggested that H(D) atoms binding to the 2nd Si layer rather than to the 3rd one in bridge positions can explain the metallization^33^. However, this study falls short in explaining the experimental data also including previous IRAS^23^,^24^ and core-level and valence-band SR-PES^7^,^25^,^26^,^30^ experiments.

### Synchrotron radiation-based photoemission measurements of the DOS

**Figure 5:**
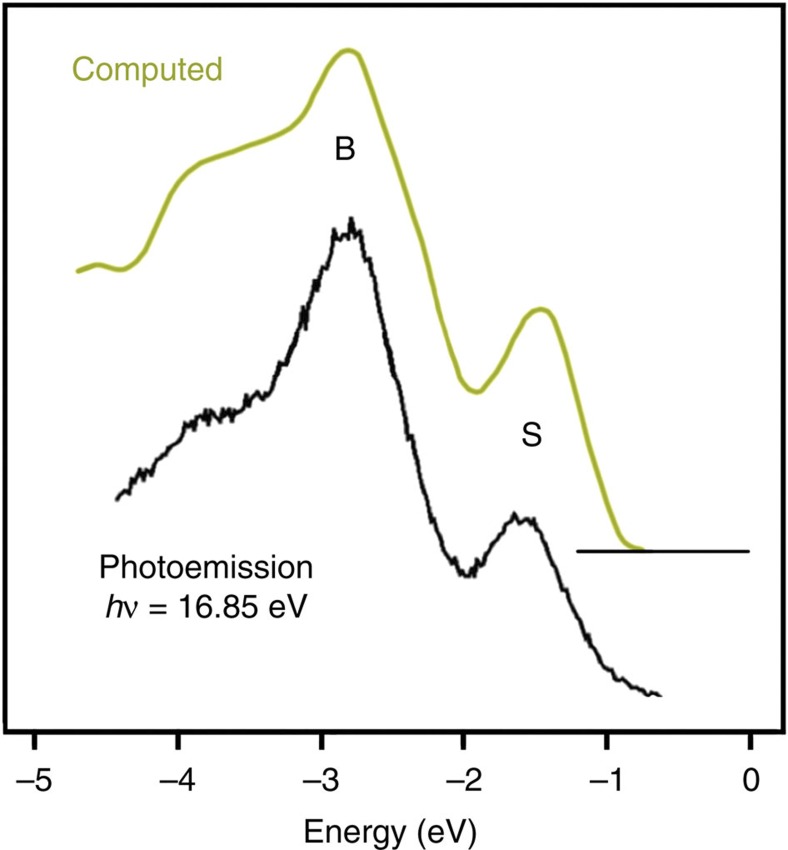
Comparison between computed and measured DOS for clean 3C-SiC(001)-3 × 2. The calculated DOS for the clean surface is compared, with the experimental valence band photoemission spectrum of 3C-SiC(001)-3 × 2 taken from ref. 23 at a photon energy of *hν*=16.85 eV. B and S stay for bulk and surface contributions, respectively. Note the excellent agreement between theory and experiment, in particular for both the shape and energy position of the bulk (B) and surface (S) electronic states.

**Figure 6:**
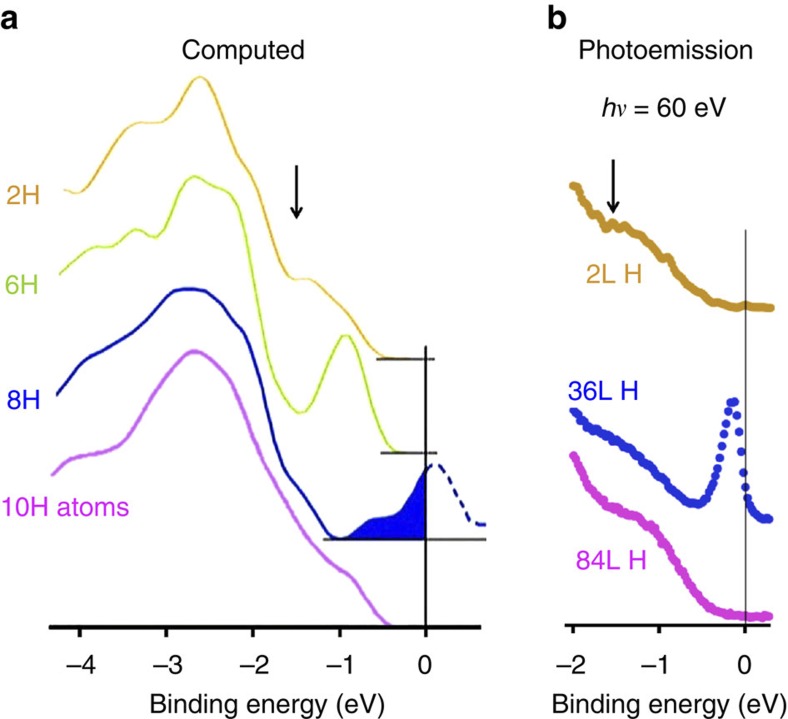
SR-PES and computed DOS for H-covered 3C-SiC(001)-3 × 2 surfaces. (**a**) Computed DOS for the 2 H, 6 H, 8 H atom and 10 H atom structures integrated over the top three Si layers and the first C layer – See Fig. 2. (**b**) SR-PES spectra recorded at a photon energy of *hν*=60 eV in the Fermi level region for 2L H exposure (semiconducting), 36L H exposure (metallic) and 84L H exposure (semiconducting) surfaces. Note the metallic–semiconducting transition upon higher H exposures in agreement with the calculated densities of states. Arrows denote the position of the surface state for the clean SiC surface, which is quenched upon H adsorption. Here also, we can note the remarkable agreement between experimentally measured DOS and computed ones.

We now focus on the corresponding electronic properties as measured by SR-PES and computed DOS for the clean and H-exposed 3C-SiC(100)-3 × 2 surface, see Figs 5 and 6, respectively. The SR-PES spectrum from the bare surface^23^ shows two dominant features labelled ‘B’ (related to bulk states) and ‘S’ (surface state related to the Si dangling bond)—see Fig. 5. The computed DOS (Fig. 6a) is integrated over the top three Si layers and the first C layer for the 2 H, 6 H, 8 H and 10 H structures. The photoemission spectra recorded at photon energy of *hν*=60 eV (Fig. 6b), in the Fermi level (*E*_F_) region for 2L H exposure (semiconducting), 36L H exposure (metallic) and 84L H exposure (semiconducting) surfaces are in excellent agreement with the calculated DOS for the 2 H, 8 H and 10 H structures, respectively (Fig. 6a).

The DOS for the clean surface is close to zero at *E*_F_ and the system is non-metallic as expected for a wide band-gap semiconductor as can be seen in the corresponding experimental and computed DOS in Fig. 6 (note the excellent agreement between the computed and experimentally measured DOS). Next, we now look in Fig. 6 at the effect of H adsorption. As can be seen in Fig. 6a,b, H eliminates already the surface state peak ‘S’ of the clean surface in the valence band at a very low 2L H exposure as also found in the computed DOS, with the corresponding structure 2 H remaining semiconducting having H atoms bonded to the first (Si1a) and second (Si2b) atomic layers. Upon further H deposition, H atoms bond to the third atomic layer (Si3a) leading to a 8 H structure exhibiting a half-filled peak at *E*_F_. The latter originates from the Si3a atom dangling bonds (see the computed DOS for the 8 H structure in Fig. 2) and corresponds to a metallic behaviour as shown in Fig. 6. Indeed, the photoemission spectrum shown in Fig. 6b, corresponding to a 36L H-exposed SiC surface, exhibits a sharp spectral feature with a clear Fermi level indicating the metallic character of this structure, in excellent agreement with the calculated DOS for 8 H structure. Saturation of the Si3a dangling bonds eliminates these mid-gap states, leading the system back to a semiconducting status as seen for the 10 H atom structure corresponding to the 84L H exposure (Figs 1, 2 and 6). Thus calculations and experiments are in perfect agreement.

Since the synchrotron beam diameter is 300 μm with photoelectrons collected from an entire area covering a surface seven orders of magnitude larger than probed by STM/scanning tunneling spectroscopy (150 × 300 Å)^23^, evidencing H-induced surface metallization by SR-PES indicated that the nanotunnel metallization extends to a large surface. The strong metallic character shown in the 36L H spectrum through intense DOS and sharp Fermi level is clearly inconsistent with short and scarce nanotunnels, but in agreement with nanotunnels covering the whole surface (Fig. 6).

## Discussion

The above results show a feature of central importance, namely the first evidence of self-organized opening of nanotunnels in a material. This is initiated by atomic H(D) bonding to the Si2a atoms, causing an inversion of the tetrahedral arrangement (puckering), thus breaking Si2a–Si3b bonds leading to highly reactive Si3a atoms. The opening of the nanotunnel frees the access to the Si3a atoms and additional H(D) atoms can form Si3a-H(D) bonds in a strongly exothermic reaction.

**Figure 7:**
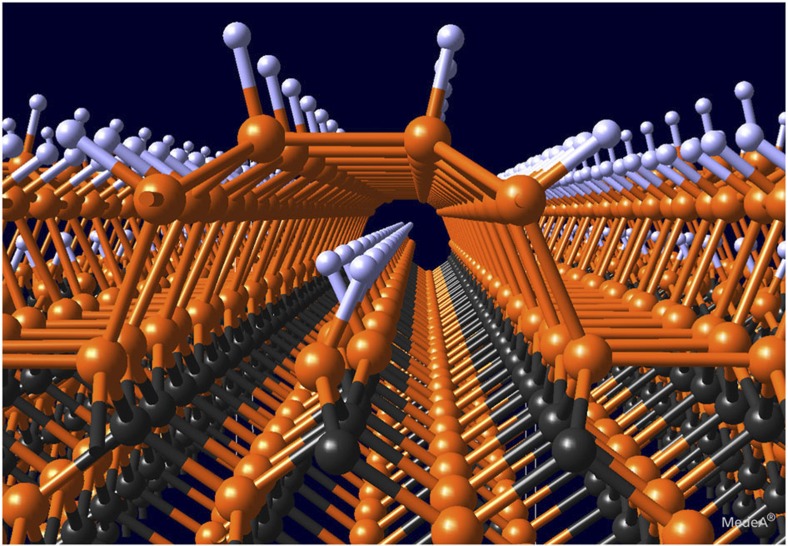
A computed view of a 3D nanotunnel. The nanotunnel opening induced by the interaction of H-atoms with the 3C-SiC(100)-3 × 2 surface is represented for the 8 H metallic structure. Note that, in the nanotunnel, the dangling bonds not terminated by a hydrogen atom are not shown.

At this stage, if the atmosphere contains other reactive atoms or chemical precursors, one can functionalize this subsurface layer exploiting the nanotunnel. In the present work, the surface is exposed only to a simple element, hydrogen, eventually leading to a fully hydrogenated structure. In the intermediate step, illustrated as a 3D view in Fig. 7, this leaves reactive dangling bonds inside the nanotunnel, which could be used to trap other species of interest.

After nanotunnel opening, the computed properties of the three-nanotunnel structures are in remarkable agreement with a range of experimental data and there is no experimental evidence, which would be in contradiction to the properties computed for these unprecedented void nanostructures. Specifically, the points are as follows: (i) Agreement with HREELS: The computed vibrational frequencies for the nanotunnel 8 H structure with both H and D atoms are in very good agreement with HREELS data as given in Table 1 and illustrated in Fig. 4. The anti-symmetric stretch mode of the surface dimers as well as the out-of-plane wag mode of the Si2a–H(D) atoms are very well described. The computed Si2a–H(D) stretch mode is not observed, which is reasonable because this motion is mostly in plane and thus expected to be weak in HREELS experiments. All experimentally observed frequencies associated with H(D) are accounted for; (ii) Agreement with IRAS: The computed Si3a–H(D) stretch modes are higher in frequency by 27 cm^−1^ compared with 22 cm^−1^ measured in infrared spectroscopy. As suggested earlier^23^, the stretch frequency of a Si–H bond should be higher if the Si atom is bonded to a C atom rather than another Si atom. The computed frequencies for the 8 H nanostructure correctly capture this situation; (iii) Agreement with SR-PES: The present calculations show that H atoms eliminate the peak originating from the asymmetric dimer on the clean 3C-SiC(100)-3 × 2 surface in agreement with the experiments. The DOS of 8 H nanostructure reveals a half-occupied narrow band at the Fermi level, which is associated with the observed metallic behaviour of this surface. Further exposure to H would fill this narrow band and the system returns to a non-metallic state, in agreement with the experimental observations. Furthermore, the above picture of H(D) atoms interacting with the third Si layer bonded to the first C plane is also consistent with previous core level SR-PES experiments indicating selective H(D) atoms interaction with the third Si layer^26^,^30^.

These nanotunnels open a direct path for the H(D) atoms towards the third Si atomic plane below the topmost surface dimers, while impeding the access to the Si dimers located in the third layer as initially assumed^23^. The ability to engineer semiconducting and/or metallic nanotunnels with such a novel one-dimensional void template offers tracks for selective interaction between dangling bonds generated inside the nanotunnel and inorganic or organic molecules, as well as metal atoms. After the puckering of the subsurface atoms, the ‘hole’ above the activated third-layer Si atoms is relatively large, namely 6.2 Å long and over 4.4 Å wide (Fig. 1). Considering the covalent radius of 1.11 Å of silicon atoms, the opening provides access to the nanotunnel for a large variety of atoms or molecules, since H is the smallest atom with atomic and covalent radii at 0.53 and 0.31 Å, respectively, leaving enough room for other atoms. Indeed, looking at the periodic table, we can see many atomic radii are smaller than 2 Å (Mendeleev periodic table, WebElements, http://www.webelements.com/). In these views Cs, one of the largest atoms used, for example, in insertion compounds as graphite^41^ is an interesting test case. So, for many atoms, there is little steric hindrance for insertion into the nanotunnel. Regarding molecules, there are many organic and inorganic molecules and radicals that have size ranging below or near 4.4 Å, small enough to make them also suitable for insertion/storage within a nanotunnel – among others one can mention OH, O_2_, N_2_, CO, CO_2_, H_2_O, C_2_H_2_, C_2_H_4_, ethylene oxide, CH_4_, NH, NH_2_, NH_3_ and many more, which may be able to pass the window above a nanotunnel (Molecular Sieves, http://chem.rochester.edu/~nvd/molecularsieves.html.). The critical geometric aspect is the 3.04 Å distance between atoms Si2a and Si3a. The chemistry of insertion needs to be chosen such that there is a strong bond between the atom Si3a and the inserted atoms or molecules while pushing atom Si2a further away from the nanotunnel.

Actually, it has already been demonstrated that a pre-oxidized 3C-SiC(100)-3 × 2 surface could be metallized by hydrogen atoms, and vice-versa, that the metallization by H atoms of a 3C-SiC(100)-3 × 2 is not removed by oxygen^7^,^23^,^24^,^25^. This leads at the same time to a surface with two opposite functionalization, metallization and passivation, a feature of very strong interest for interfacing with biology. Since the only remaining available reactive sites at the hydrogenated 3C-SiC(100) surface are the Si dangling bonds located within the 6 H and 8 H nanotunnels (all the other ones in the first and second layers being decorated by H atoms), this clearly indicates that the oxygen molecule/atom could go only into the nanotunnel. In addition, infrared absorption vibrational spectroscopy (IRAS) measurements have shown that the H–Si (2,118 cm^−1^) and H–Si–C (2,140 cm^−1^) bonds are not affected by oxygen exposures of the H–3C–SiC(100)-3 × 2 surface^24^. Therefore, one can safely anticipate that the same situation will occur with other molecules.

Another interesting test case would be a metal atom like Cs, a ‘huge’ atom which, despite its size, is very well known to intercalate easily below a surface like graphite, passing through the first atomic layer leading to alkali metal-intercalated graphite compounds^41^. Indeed, the atomic and covalent radii of a Cs atom are 3 and 2.44 Å, respectively (Mendeleev periodic table, WebElements, http://www.webelements.com/), making them very suitable to insert within a ≥4.4 Å nanotunnel having empty Si dangling bonds, which once again, are the only available ‘reactive’ adsorption sites. It is very well known that a polarized covalent bonding through hybridization between the alkali ‘s’ spherical orbitals with localized ‘p’ electronic states is taking place – as shown, for example, for Si^42^. In addition, one should mention that Cs and other alkali metals are excellent catalysts, like, for example, of oxidation and nitridation, opening very interesting prospects towards selective surface functionalization^42^. Finally, alkali metals are known to lower significantly the surface work function, which could also be a very useful feature too.

Placing metal atoms within the nanotunnels, one could also tune the Schottky barrier height to engineer the Fermi level (*E*_F_) pinning position. Alkali metals have indeed been shown to selectively pin the Fermi level for other compound semiconductors such as III–V materials like InAs, GaAs, InP or GaSb^43^,^44^,^45^,^46^, with *E*_F_ coming thereby at the highest energy level position above a semiconductor conduction band minimum as, for example, for Cs/InAs^43^. Adsorption of alkali metals have also other interesting properties such as their ability to control and engineer the line-up at heterojunctions and insulator/semiconductor interfaces^47^.

Other metals such as Ga or Pb could also be used as also shown, for example, for GaAs^48^. Furthermore, inserting full d-band or jellium-like metals within the nanotunnel, it would potentially be possible to set the Schottky barrier height upon request, depending on the selected species as shown for, for example, with Au or Al on a II–VI semiconductor^49^ – Note that the atomic and covalent radii for Ga are: 1.36 and 1.22 Å, for Pb: 1.54 and 1.46 Å, for Au: 1.74 and 1.34 Å and for Al: 1.18 and 1.21 Å, respectively (Mendeleev periodic table, WebElements, http: //www.webelements.com/.), that is, once again, well below the 6.2 × 4.4 Å hole’s aperture above the nanotunnel (Fig. 1b).

It is interesting to note that porous molecular organic nano-cages have been built as ’host molecules’ that exhibit a selective interaction with incoming ‘guest molecules’^50^. Also, supra-molecular cages assembled with H atoms, have been shown to encapsulate molecules or metal complexes^51^,^52^. Similarly, molecules could be let into the nanotunnels either from the surface through the 6.2 × 4.4 Å hole’s aperture above the nanotunnel, or alternately, at a step edge, where the nanotunnels begin or end. The entrance could be found by shooting them directly in exploiting the directionality of a supersonic molecular beam in which the molecules are carried by an inert gas. As recently shown, symmetric tops can be aligned with respect to the rotational axis, increasing significantly the probability of entering with the molecular axis oriented upon purpose, either along or from the top, perpendicularly to the nanotunnel axis^53^,^54^,^55^.

We can thus envisage using such nanotunnels as nanotraps with intercalation of guest atom/molecule. Indeed, intercalation of atoms as large as Cs and even of C_60_ molecules (having a 7 Å diameter) under a graphene sheet has been reported^56^,^57^. Last but not least, the trapping of guest atoms can be used to tailor the electronic properties of the nanotunnels in analogy to what happens for SWCNT (single-walled carbon nanotubes) for which the size of the intercalated atom has been demonstrated to play a key role^58^. The SWCNT small inner diameter (1.75 Å) allows iodine ion insertion leading to selective doping^58^. Once again, this indicates that a similar insertion should not be a problem with a nanotunnel having a 4.4 Å width and a 6.2 Å top access and 1 (8 H) or 2 (6 H) dangling bonds (Fig. 1).

Therefore, insertion into the nanotunnels of most of these atoms or molecules could even take place through intercalation below a graphene layer grown or transferred onto a 3C-SiC(001)-3 × 2 nanotunnel surface, offering very interesting possibilities and opportunities to monitor the graphene/SiC interface including doping and its resulting properties, Indeed, a graphene sheet could be transferred on top of these nanotunnels^59^ enabling atomic scale heterojunctions or Schottky barriers formation.

Interestingly, graphene layer growth can also be achieved using a microwave plasma technique at low substrate temperatures (300 °C instead of >1200 °C using other processes)^60^. This offers another promising alternative, opening engineering capabilities for graphene/nanotunnel interface formation without hydrogen atoms removal from the SiC surface/subsurface or nanotunnel damage. Indeed, not only metal atoms like alkalis and other metals could go below the graphene layer (as in graphite surface), but also inorganic or organic molecules too, and interact with the nanotunnels as shown for, for example, for carbon nanotubes^58^.

There is a large variety of new physics and chemistry that could be used to functionalize such a SiC subsurface modified by hydrogen atoms, triggering interesting research directions, in particular in the field of graphene substrate’s functionalization. It impacts important issues such as tunneling nano-junctions that are of interest in electronics/spintronics applications. Using the nanotunnels for ‘subsurface wiring’ with vertical molecular connections to the outside could potentially be possible and presents another perspective that could be explored. Furthermore, compounds other than SiC showing the combination of surface and chemically induced puckering of subsurface atoms could lead to systems with larger nanotunnel dimensions. So, these findings could be useful in opening-up a class of promising nano-materials having a broad field of potentially interesting engineering capabilities.

## Methods

### Details about SiC samples and hydrogen exposures

We use high-quality single-domain single-crystal 3C-SiC(001) few μm thin films grown on a vicinal (4°off) Si(001) substrate by NovaSiC and CEA-LETI. Low (10^16^ atoms cm^−3^) and medium (10^17^ atoms cm^−3^) *n*-doped samples were used for HREELS and SR-PES, respectively. Note that, with these two SR-PES and HREELS techniques, the information is collected in a 7 × 10^+4^ μm^2^ area. HREELS and SR-PES experiments are performed at a base pressure lower than 2 × 10^−10^ mbar*. In situ* atomic H exposures are performed with high-purity research-grade molecular H_2_ introduced by a capillary tube leading to the SiC sample with its surface maintained at 300 °C and dissociated by a heated tungsten filament. To avoid contamination, thorough baking of the gas lines and outgassing of the tungsten filaments are performed. The absolute determination of H coverage for experiments performed in different laboratories is not straight forward since it depends critically on the efficiency of the H_2_ cracker. Then, the H atoms react with the 3 × 2 surface reconstruction of the 3C-SiC(001)-3 × 2 surface (which is inert to molecular H_2_). However, after dissociation, some of the H atoms recombine to re-form H_2_. This recombination process depends very much on the geometry of the vacuum chambers, which are very different in the HREELS and the photoemission experimental systems. So, contrary to other adsorbed species, the problem with atomic H experiments is that, the exposure is known, not the exact coverage. Here, the 36L H PES (Synchrotron SOLEIL Tempo beamline) and the 50L H HREELS (IMEM-CNR & University of Genoa joint laboratory) correspond both to the H-coverage leading to the metallization, that is, to the calculated 8 H metallic structure, so they are comparable. All other details, in particular about sample preparation and atomic H/D exposures are available elsewhere^7^,^8^,^9^,^10^,^23^,^25^,^26^,^30^.

### HREELS experiments

**Figure 8:**
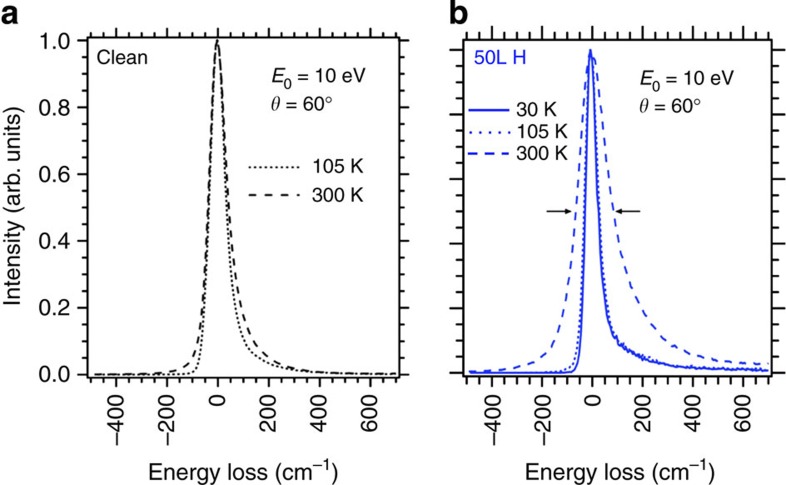
HREELS specular elastic peak. The elastic peak is measured for different crystal temperatures (30, 105 and 300 K) for (**a**) clean and (**b**) hydrogen-covered 3C-SiC(001)-3 × 2 surface under identical experimental conditions and spectrometer settings. It shows the subsequent peak broadening upon a 50 L hydrogen exposure. See also at the ‘Vibrational properties measured by HREELS’ section in ‘Results’.

The HREELS measurements were carried out in Genoa using a Delta 0.5 SPECS spectrometer in-specular and out-of-specular, at a scattering angle *θ*=60° from the surface normal, with primary electron beam energies *E*_0_=2.5 and 10 eV at 4 meV energy resolution. The accuracy in vibrational frequencies determination is, however, better and estimated at ±1 meV, that is, ±8 cm^−1^. The data are collected at room and low temperatures. The achievement of the metallic state is evidenced in the HREELS data through the elastic peak broadening as already shown above in Fig. 3 and also in Fig. 8 for the clean and the 50 l hydrogen exposed 3C-SiC(001)-3 × 2 surfaces, respectively, at 30, 105 and 300 K. We notice that the specular peak broadens with temperature in both cases, and that the effect is much stronger for the H-covered case. This phenomenon has been investigated by Persson and Demuth and published in a seminal paper in 1984^38^. In particular, they solved analytically the cases corresponding to: (a) a doped semiconductor: Applying the formula given in ref. 38 to the bare surface, we obtain a carrier density of 1,017 carriers per cm^3^ which compares reasonably well with the nominal doping of our sample of ≈1,016 carriers per cm^3^; and to (b) a metallic film on a semiconducting surface. This case was solved for the limits in which, the plasmon energy is much smaller and much larger than thermal energy at room temperature. These limits are verified for the cases of Si(111)-7 × 7 (plasmon energy *ħω*_p_=0.2 meV) and for ultrathin films of Au and Pd on Si(111) (*ħω*_p_>>kT). In the first case, the broadening is due to the multiple excitation of the plasmon, whereas in the second case, it is associated to generation of electron hole pairs (Drude damping). For Si(111)7 × 7 the carrier density is 1/49 per substrate unit cell and the effective mass of the electron 60 free electron masses. For 8 H-3C-SiC(001)-3 × 2, the carrier density is ~15 times larger. Assuming a unitary effective mass, we get an upper limit for the plasmon energy of 6 meV, which is in between the limits evaluated by Person and Demuth^38^. A quantitative evaluation of the broadening is therefore not possible, but one can safely derive that the observed temperature-dependent broadening indicates a metallic behaviour.

### Synchrotron radiation-based PES experiments

Valence-band SR-PES experiments are performed at 300 K, using the ultrahigh vacuum photoemission experimental station of the TEMPO beam line connected through an ondulator to the light emitted by the third generation synchrotron radiation SOLEIL light source. The diameter of the SR beam and the size of the photoelectrons collected area are 300 μm. Photoemission spectra are measured using a Scienta SES2002 hemispherical electron energy analyser equipped with a fast two-dimensional delay line detector. The overall energy resolution combining the contributions of the soft X-ray beam monochromator and analyser energy selection is, in our experimental conditions, better than 50 meV.

The experimental station allows direct *in situ* atomic H exposures during photoelectron spectroscopy data acquisition up to partial pressures of 10^−8^ mbar. Particular care was taken in protecting the sample surface from metallic contamination of the surface induced by the high temperature tungsten filament used to dissociate the H_2_ molecules.

The achievement of the metallic state is evidenced through the real-time measurement of the Fermi level kinetic energy region and by monitoring the corresponding build-up of the DOS as presented in Fig. 4.

### *Ab-initio* simulations

The interaction of H atoms with this surface is investigated also by *ab-initio* calculations on a model, which consists of five Si layers and four C layers. The top layers represent the (3 × 2) reconstructed surface. For convenience, the bonds of the carbon layer at the bottom of the model are saturated with selenium atoms. The calculations are based on DFT^61^,^62^ with the generalized gradient approximation in the form proposed by Perdew, Burke and Ernzerhof^63^. The Kohn–Sham equations are solved with the all-electron frozen-core projector augmented wave method^64^ as implemented in the Vienna *ab-initio* Simulation Package^65^,^66^,^67^ and integrated in the MedeA® computational environment^67^. Locally stable equilibrium structures are determined by minimizing energy and forces using a conjugate gradient method. The topmost six layers are relaxed so that the forces on the atoms are <0.02 eV Å^−1^. The three layers at the bottom are kept frozen. The vibrational frequencies are computed using a finite difference method^68^ as also implemented in the MedeA^®^ environment^67^. In these phonon calculations, the bottom layers are kept frozen and phonon dispersions originating from the vibrations of all other atoms are computed. For the clean 3C-SiC(001)-3 × 2 surface, comparison between computed DOS and measured one by valence band photoemission is shown in Fig. 8, with the valence band photoemission spectrum taken from ref. 23. Note the remarkable agreement between theory and experiment, in particular for the shape and energy position of the bulk (B) and surface (S) electronic states.

## Author contributions

P.S., E.W. and M.R. conceived and conducted the research project, and wrote the paper. E.W. carried out all calculations. E.C., C.G., S.B., L.V., L.S., P.S. and M.R. performed the HREELS experiments. A.T., M.S., M.D’a., F.S. and P.S. performed the SR-PES experiments. All the authors participated in the data analysis and general discussions, and commented on the manuscript.

## Additional information

**How to cite this article:** Soukiassian, P. *et al.* Hydrogen-induced nanotunnel opening within semiconductor subsurface. *Nat. Commun.* 4:2800 doi: 10.1038/ncomms3800 (2013).

## Figures and Tables

**Table 1:** Measured and computed vibrational frequencies of the metallic structure.

**Hydrogen**				**Deuterium**			
**Theory (cm**^**−1**^**)**	**Infrared (cm**^**−1**^**)^23^**	**HREELS in specular (cm**^**−1**^**)**	**HREELS out of specular (cm**^**−1**^**)**	**Theory (cm**^**−1**^**)**	**HREELS in specular (cm**^**−1**^**)**	**HREELS out of specular (cm**^**−1**^**)**	**Motion occurring in the vibrational mode**
600		635	600	438	Not observed	435	Wagging Si(2a)
621				422			Wagging Si(1a)
2,120	2,140			1,530			Stretch Si(3a)–H(D)
2,093 (2,115)	2,118	2,130		1,511 (1,527)	1,540		Stretch Si(1a)–H(D)
2,087 (2,110)			2,120	1,506 (1,523)		1,532	Stretch Si(1a)—H(D) antiphase
2,020		Not observed		1,457	Not observed		Stretch Si(2a)—H(D) in plane

The vibrational frequencies measured (50L H(D)-exposed) and computed (8 H(D)) for the metallic structure (see Fig. 1) are shown above. Frequencies are in cm^−1^. The modes are strongly coupled. The HREELS data (50L H/D) recorded out of specular are sensitive also to the antiphase motion of the H(D) atoms bonded to first and third Si layer, while only the symmetric modes can be excited in-specular. The computed values in parentheses for the Si(1a)–H(D) stretch mode are for a structure with H(D) atoms bonding only to Si1a atoms of the surface dimer.
